# Supplementary material for: Bubble Formation in Pulsed Electric Field Technology May Pose Limitations
Source: Micromachines (Basel). 2022 Jul 31;13(8):1234. doi: 10.3390/mi13081234 (PMC9414362; doi:10.3390/mi13081234)
Supplement: Supplementary file 1 [file micromachines-13-01234-s001.zip › micromachines-1754161-supplementary.pdf]

Table S1. Bubble diameter evolution as a function of time. Measurements at 180° of the electrode.

time [ms]	100 V 180°		60V 180°		30V 180°	
	Av. Bubble diameter [um]	yEr +-	Av. Bubble diameter [um]	yEr +-	Av. Bubble diameter [um]	yEr +-
0.000	0.000	0.000	0.000	0.000	0.000	0.000
0.100	0.855	0.855	2.849	0.205	0.000	0.000
0.200	3.846	2.248	9.117	0.285	0.000	0.000
0.300	6.410	2.824	11.396	0.317	5.128	--
0.400	8.547	3.347	17.094	0.430	6.268	0.570
0.500	13.248	1.895	19.943	0.390	9.117	0.570
0.600	19.658	2.151	25.641	0.690	11.396	0.570
0.700	20.940	2.552	39.316	1.130	13.105	1.140
0.800	23.932	3.042	42.735	1.350	13.105	0.570
0.900	23.932	3.626	44.444	1.381	14.815	0.570
1.000	25.641	4.009	45.014	1.386	15.385	0.987

Table S2. Bubble diameter evolution as a function of time. Measurements at 90° of the electrode.

time [ms]	100 V 180°		60V 180°		30V 180°	
	Av. Bubble diameter [um]	yEr +-	Av. Bubble diameter [um]	yEr +-	Av. Bubble diameter [um]	yEr +-
0.000	0.000	0.000	0.000	0.000	0.000	0.000
0.100	3.632	2.525	3.704	0.754	0.000	0.000
0.200	11.325	3.408	6.553	1.027	1.709	0.987
0.300	20.085	4.904	12.536	0.570	3.946	2.043
0.400	28.419	7.672	15.954	2.484	6.268	2.484
0.500	39.316	8.632	18.803	1.974	9.687	2.484
0.600	42.949	8.156	22.222	3.419	10.826	2.484
0.700	46.581	8.465	26.496	4.274	12.536	2.484
0.800	52.991	7.210	32.479	2.961	18.234	1.508
0.900	53.846	7.972	31.909	3.015	18.234	3.466
1.000	53.846	7.661	32.479	3.558	20.513	3.558

Table S3. Bubble diameter evolution as a function of time. Measurements at 0° of the electrode.

time [ms]	100 V 180°		60V 180°		30V 180°	
	Av. Bubble diameter [um]	yEr +-	Av. Bubble diameter [um]	yEr +-	Av. Bubble diameter [um]	yEr +-
0.000	0.000	0.000	0.000	0.000	0.000	0.000
0.100	13.248	5.937	4.516	1.500	3.989	2.054
0.200	27.350	5.175	14.815	2.849	7.977	1.140
0.300	40.171	5.860	23.932	0.987	12.536	2.054
0.400	53.419	4.953	31.339	1.508	15.385	2.611
0.500	62.821	7.198	37.607	1.709	21.083	2.054
0.600	71.795	8.855	45.584	2.279	21.652	2.054
0.700	85.470	8.882	58.689	8.218	34.188	5.222
0.800	98.291	8.786	71.795	11.382	35.328	4.967
0.900	111.111	9.992	77.493	11.093	34.188	6.908
1.000	111.966	9.479	76.923	4.523	35.328	8.277
